# Endovascular Treatment of Patients with Ruptured Intracranial Aneurysms: A Series of 468 Patients Treated Over a 14-Year Period

**DOI:** 10.5334/jbsr.2550

**Published:** 2022-03-28

**Authors:** Franny Hulscher, Benjamin Mine, Stéphanie Elens, Thomas Bonnet, Juan Vazquez Suarez, Boris Lubicz

**Affiliations:** 1 Hopital Erasme, BE

**Keywords:** intracranial aneurysm, SAH, neurointerventional, endovascular treatment, ruptured aneurysm

## Abstract

**Purpose:**

Non-traumatic subarachnoid hemorrhage (SAH) is an emergency usually caused by the rupture of a saccular intracranial aneurysm. Endovascular treatment (EVT) is now considered as the first therapeutic option. The aim of our study is to evaluate, over a 14-year period in a single center, the result of EVT of ruptured intracranial aneurysms.

**Methods:**

From the retrospective analysis of our prospectively maintained database, we collected data of 457 patients successfully treated by endovascular approach for a SAH. Descriptive statistics and percentages were used to report clinical and anatomical outcomes, procedure-related complications, post procedural events, morbidity and mortality.

**Results:**

EVT was unsuccessful in eleven patients but effective in 457 patients with two patients who experienced a rebleeding (0.4%). In 6.3% of cases, a second EVT was necessary. The final aneurysm occlusion was complete (65.7%), with a neck remnant (28.2%) or incomplete (6.1%). Procedure-related complications occurred in 5.9% of patients and were associated with five clinical worsening and one death. Overall EVT-related morbidity and mortality were thus of 1.3% and 0.4% respectively. At discharge, 71% of patients had a good recovery (mRS 0–2), 11.2% had a poor outcome (mRS 3–5), and 17.8% died.

**Conclusion:**

This study seems to prove that high-volume centers with experienced interventional neuroradiologists carry low rates of technical failure and complication from EVT of ruptured intracranial aneurysm.

## Introduction

Non-traumatic subarachnoid hemorrhage (SAH) is a major life-threatening emergency. In 80% of cases, it is caused by the rupture of a saccular intracranial aneurysm (sIA). Other common causes are dissecting aneurysms, cerebral arteriovenous malformations and vasculitis [1,2,3].

The hallmark symptom is a sudden and severe headache. Associated signs include nausea, vomiting, photophobia, neck stiffness, focal neurologic deficits, seizure or depressed consciousness [1,2,4]. The initial clinical severity is determined by simple validated grading system like the World federation of Neurosurgical Societies (WFNS) that is the most used indicators and considered as a major determinant of the prognosis [4,5].

SAH may cause acute hydrocephalus and brain edema. Later complications include vasospasm and delayed cerebral ischemia that are associated with serious damages even after the aneurysm treatment [1,2,5].

Aneurysmal SAH are most often treated within 24 – 72 hours [3,4,5]. Neurosurgical clipping and endovascular treatment (EVT) by endosaccular coiling are both effective for the treatment of saccular intracranial aneurysms. These treatments have been compared and EVT is now considered as the first therapeutic option in most cases. Treatment choice is made by a multidisciplinary team including interventional neuroradiologists (INR), neurosurgeons, intensivists, and neurologists [3,4,5,6,7,8,9].

The aim of our study is to evaluate, over a 14-year period in a single high-volume center, the results of EVT of ruptured intracranial aneurysm.

## Patients & Methods

### Study design and patients

This study was approved by our institutional ethical committee (n°P2019/152). Our prospectively maintained database was retrospectively analyzed to identify, between April 2004 and June 2018, all patients treated only by endovascular approach for a ruptured IA.

### Collected data

Available data were collected from the admission date in different institutions to collect the first bleeding time. The outcomes of our patients were followed until they were discharged from our hospital, or another medical institution and no clinical results were collected beyond three months of follow-up after EVT.

### Endovascular procedure and external ventricular drain

**Figure 1:**
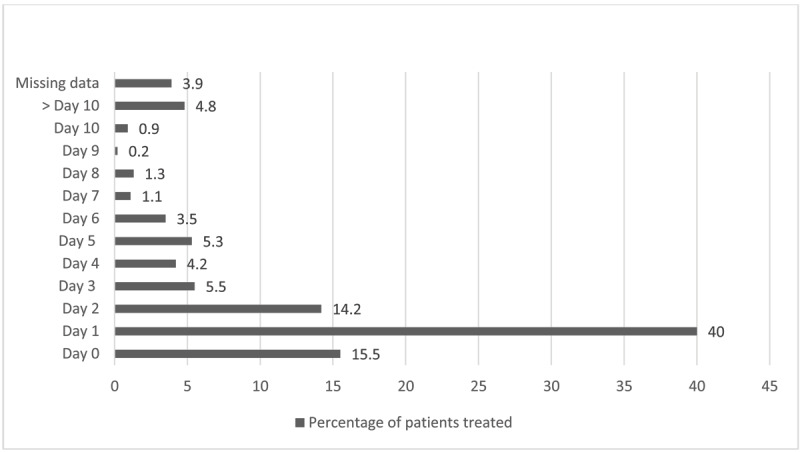
Percentage of patients treated according to the day following the SAH.

All EVT were performed by an INR from our institution. As it is reflected in ***Figure 1***, the majority of patients were treated within the first days following the SAH (median = day 1 and interquartile range = 2 days).

Placement of an external ventricular drainage (EVD) was decided by our neurovascular team. Patients were then monitored in our intensive care unit until the overall stabilization of their condition.

### Statistical analysis

The sample was analyzed by descriptive statistics. Quantitative data were expressed in mean values ± standard deviation (SD) or medians and 95% confidence intervals (CI) or interquartile range, accordingly, after verification of normality of distributions by the Kolmogorov-Smirnov test. Qualitative data were expressed by the way of percentages.

### Patient characteristics

Four hundred sixty-eight patients were identified. In eleven patients, there was a failure of EVT (2.4%). These patients were excluded from the present analysis and are detailed in Appendix.

Our final cohort includes 457 patients successfully treated by endovascular approach. Patient characteristics are detailed in ***Table 1***.

**Table 1:** Patient characteristics (n = 457). Abbreviations as in the text.


AGE *(YEARS)*	52 ± 14.2 (SD)

**Gender**	

Male	169 (37%)

Female	288 (63%)

**WFNS** *(before the first procedure)*	

Grade 1	231 (50.6%)

Grade 2	75 (16.4%)

Grade 3	8 (1.8%)

Grade 4	81 (17.7%)

Grade 5	62 (13.6%)

**EVD**	

Yes	192 (42%)

No	255 (58%)


Imaging characteristics of 457 patients successfully treated by endovascular approach are detailed in ***Table 2***.

**Table 2:** Imaging characteristics (n = 457). Abbreviations as in the text.


ORIGIN OF SAH (N = 457)	

Saccular aneurysm	414 (90.6%)

Fusiform aneurysm	10 (2.2%)

Dissecting aneurysm	33 (7.2%)

**Location**	

Anterior communicating complex (ACom)	189 (41.4%)

Middle cerebral artery (MCA)	65 (14.3%)

Posterior communicating artery (PCom)	79 (17.3%)

Internal carotid artery (ICA)	38 (8.4%)

Pericallosal artery	4 (1.8%)

Basilar artery tip	28 (6.1%)

Posterior inferior cerebellar artery (PICA)	17 (3.8%)

Vertebral artery	14 (3.1%)

Others	23 (5%)

**Size range of aneurysm ^a^ ^b^ (n = 424)**	

Small (<10 mm)	352 (83%)

Large (10 to 25 mm)	70 (16.5%)

Giant (>25 mm)	8 (1.9%)

**Associated lesion ^c^ (n = 457)**	

None	321 (70.2%)

Cerebral arteriovenous malformation	10 (2.2%)

**Other aneurysm**	**113 (24.7%)**

Dissection	5 (1.1%)

Carotid stenosis	3 (0.7%)

Vasospasm	13 (2.8%)

Carotid thrombosis	1 (0.2%)

Major decreased cerebral perfusion	2 (0.4%)

**Number of associated aneurysms**	Median = 1 ; 95% CI [1,2,3]


^a^ Dissection (n = 33) size was not measured. ^b^ Maximal diameter. ^c^ Before EVT.

## Results

### Procedures

**Figure 2:**
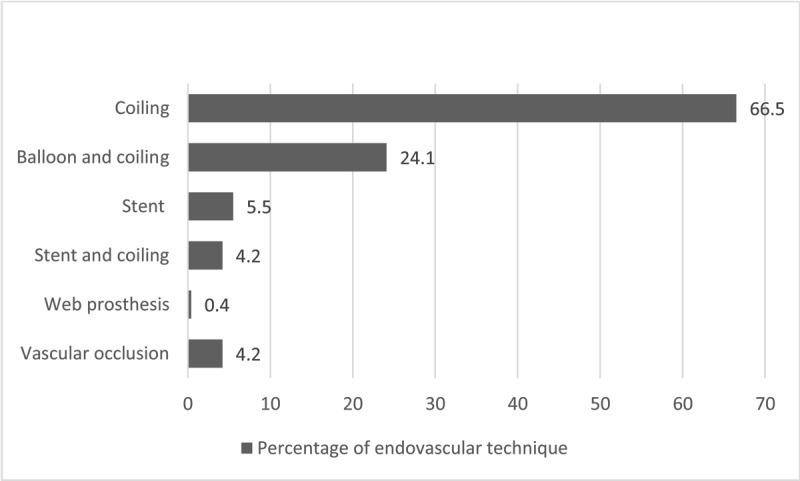
Percentage of endovascular technique used for intial treatment of the SAH.

**Figure 3:**
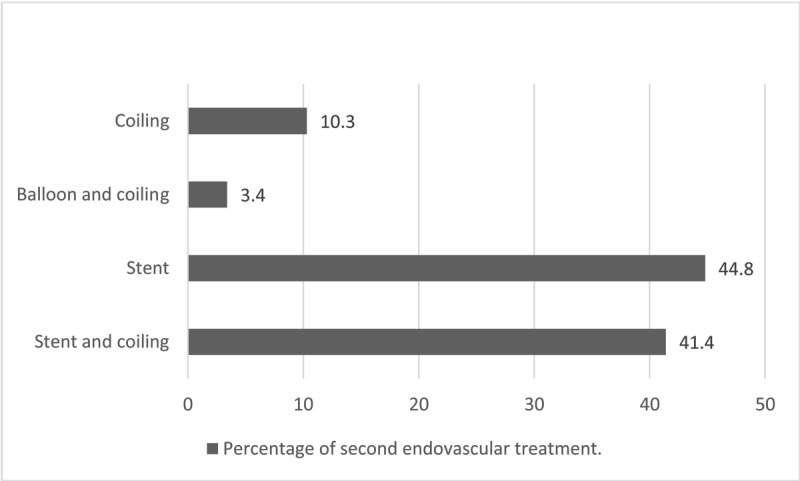
Percentage of endovascular method when a second treatment was necessary.

***Figure 2*** shows the endovascular technique used for EVT. In 6.3% of the cases (n = 29/457), a second EVT was necessary to completely exclude the aneurysm or the arterial dissection. ***Figure 3*** shows the second endovascular method.

### Anatomical outcome

Regarding aneurysm or arterial dissection occlusion, EVT achieved a complete occlusion in 65.7% of the cases. There was a neck remnant in 28.2% and an incomplete occlusion in 6.1% of the cases.

### Procedure related complications and clinical outcomes

Procedure-related complications occurred in 27 cases (5.9%) in 26 patients.

Complications included 9 thromboembolic events (2%), 6 aneurysm perforations (1.3%), 5 vasospasms (1.1%), 2 coil migrations (0.4%), 4 arterial dissections (0.9%), one WEB device migration (0.2%). These complications were associated with clinical consequences in 6 patients with 5 worsening of neurological exam and 1 death. Immediate EVT-related morbidity and mortality were thus 1.1% and 0.2% respectively.

### Immediate post-procedural Glasgow Outcome Score (GOS)

**Figure 4:**
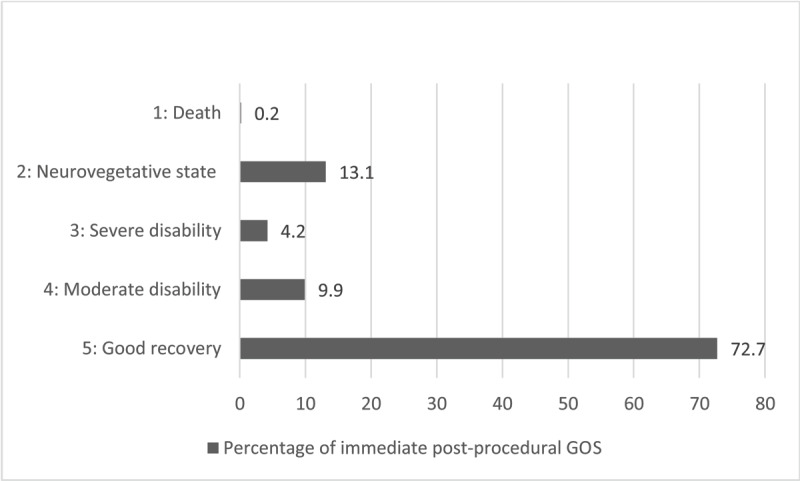
Percentage of immediate GOS after EVT.

Immediate clinical outcomes were collected within 24 hours after EVT and are detailed in ***Figure 4***.

### Post-procedural events

Clinical complications occurred in 246/457 (53.8%) patients. These events are detailed in ***Table 3***.

**Table 3:** Post-procedural events (n = 246).


**Vasospasm and delayed cerebral ischemia (DCI)**	166 (67.5%)

**Intracranial hypertension**	54 (22%)

**Epileptic seizure**	38 (15.5%)

**Ventriculitis**	37 (15%)

**Hydrocephalus**	23 (9.4%)

Stroke	12 (4.9%)

Septic shock	11 (4.5%)

Terson syndrome	9 (3.7%)

Status epilepticus	9 (3.7%)

Cardiogenic shock	5 (2%)

EVD related hemorrhage	5 (2%)

Pulmonary embolism	4 (1.6%)

Digestive ischemia	3 (1.2%)

Acute respiratory distress syndrome (ARDS)	2 (0.8%)

Meningitis	2 (0.8%)

Aneurysm rebleeding	2 (0.8%)

Transient ischemic attack	1 (0.4%)

Myocardial infarction	1 (0.4%)

Cardiorespiratory arrest	1 (0.4%)

Intra-stent stenosis	1 (0.4%)


Aneurysm rebleeding occurred in 2/457 patients (0.4%):

- A 34-year-old woman with a WFNS grade 3 and a large MCA sIA was treated by coiling on the 8^th^ day after SAH. A vasospasm was identified before EVT and the occlusion of the sIA was incomplete. The next day, a rebleeding occurred and an EVD was needed with a second EVT by stenting and coiling. The patient kept a moderate disability at discharge and a neck remnant regarding the occlusion of the sIA
- A 66-year-old man with a WFNS grade 4 and a posterior cerebral artery dissection was treated by stenting on the 2^nd^ day after SAH. The patient presented then a major vasospasm and hydrocephalus that had worsened his clinical situation. The rebleeding occurred ten days after the EVT and left him in a brain-dead state.

Overall EVT-related morbidity and mortality were thus 1.3% and 0.4% respectively.

There were 37 ventriculitis and 2 meningitis among 192 EVDs placed. Overall EVD-related infections were thus 20.3%.

### Clinical outcomes at discharge

**Figure 5:**
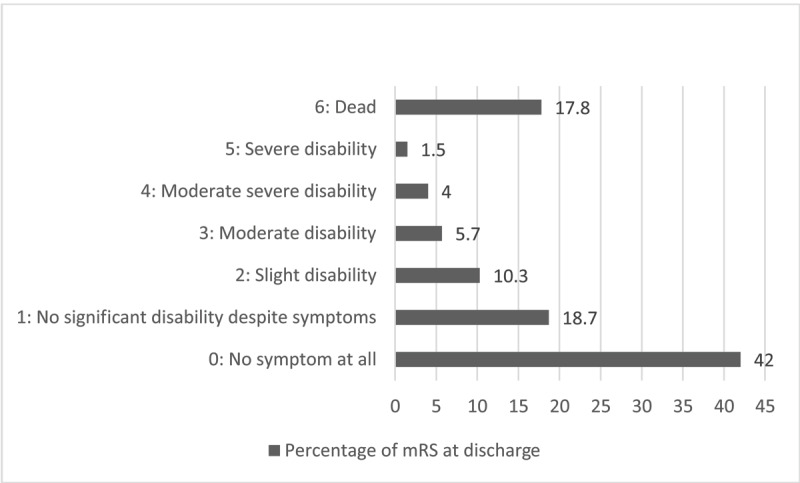
Percentage of mRS at discharge.

Modified Rankin Scale (mRS) at discharge is shown in ***Figure 5***.

**Figure 6:**
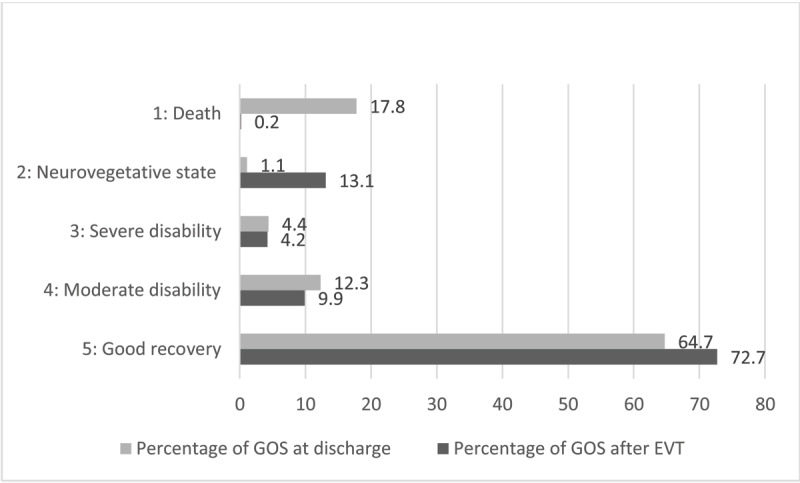
Comparison between percentage of GOS immediately after EVT (darker gray) and at discharge (lighter grey).

***Figure 6*** shows the comparison between GOS immediately after EVT (darker gray, see ***Figure 4***) and GOS at discharge (lighter grey).

The report of the clinical results (mRS) at discharge according to the initial WFNS grade is detailed in ***Table 4***.

**Table 4:** mRS according to initial WFNS. Abbreviations as in the text.


WFNS	MRS	N	%

**grade 1**	0 = No symptoms at all	154	66.7

	1 = No significant disability despite symptoms	35	15.2

	2 = Slight disability	19	8.2

	3 = Moderate disability	6	2.6

	4 = Moderate severe disability	2	0.9

	5 = Severe disability	1	0.4

	6 = Dead	14	6.1

	**Total**	**231**	**100**

**grade 2**	0 = No symptoms at all	27	36.0

	1 = No significant disability despite symptoms	20	26.7

	2 = Slight disability	7	9.3

	3 = Moderate disability	5	6.7

	4 = Moderate severe disability	2	2.7

	5 = Severe disability	2	2.7

	6 = Dead	12	16.0

	**Total**	**75**	**100**

**grade 3**	0 = No symptoms at all	0	0

	1 = No significant disability despite symptoms	3	37.5

	2 = Slight disability	2	25

	3 = Moderate disability	2	25

	4 = Moderate severe disability	1	12.5

	5 = Severe disability	0	0

	6 = Dead	0	0

	**Total**	**8**	**100**

**grade 4**	0 = No symptoms at all	10	12

	1 = No significant disability despite symptoms	20	25

	2 = Slight disability	14	17

	3 = Moderate disability	7	9

	4 = Moderate severe disability	3	4

	5 = Severe disability	1	1

	6 = Dead	26	32

	**Total**	**81**	**100**

**grade 5**	0 = No symptoms at all	2	3

	1 = No significant disability despite symptoms	7	11

	2 = Slight disability	5	8

	3 = Moderate disability	6	10

	4 = Moderate severe disability	10	16

	5 = Severe disability	3	5

	6 = Dead	29	47

	**Total**	**62**	**100**


## Discussion

### Patients and imaging characteristics

Our WFNS grades correspond to the ARETA trial and the CLARITY studies and show a similar population with most patients with a favorable grade at admission [10,11].

In this study, the proportion of saccular intracranial aneurysms (90.6%) and arterial dissection (7.2%) is probably higher because we have excluded etiologies that did not require an EVT. The most common sites of ruptured aneurysms are the ACom, the Pcom and the MCA with often unique aneurysm which are in line with our results. The median size of ruptured aneurysms is around 6 mm and most of intracaranial aneurysms are smaller than 1 cm (around 80–90% of cases) like in our study which highlights the rupture risk even with small aneurysms [2,6,7,9,10,11,14,15,16,17].

### EVT procedure and anatomical outcome

Our results show high use of intracranial stents and vascular occlusion. It can be explained by several factors: (1) a high percentage (9.4%) of dissections and fusiform aneurysms; (2) stents are more often used for larger aneurysms (18.4% in our study) and/or wide neck aneurysms (although neck size was not measured in our data).

Occlusion rates reported in our series were like the CLARITY and Park et al. studies [6,9,10,16,18,19,20,21].

### Procedure-related complications and clinical outcomes

In our study, the rates of intraoperative complications, EVT-related morbidity and mortality are lower than in the literature [19,21,22].

Regarding thromboembolic events (2% in our study), the range in the literature is between 2.5% and 28.0% [19,21,22,23]. Good results can possibly be explained by the use of a strict heparinization protocol, the same as for unruptured aneurysms. The aim is to double the activated clotting time (ACT) during EVT, and to control it every 30 minutes. Heparinization is then prolonged for 12-24h in most patients. Some studies showed comparable good results using continuous heparin for 24h without a significant increase of hemorrhagic complications [22,24].

The rate of intraoperative rupture in our study was 1.3% which is lower to the reported rates found in literature (4.4–7.6%) [19,21,23]. Practitioner experience and centers with high number of patients have lower complication rate and improve outcomes from SAH which could also explain our good results. Indeed, in our center, around 250 IA are yearly treated, most of them being unruptured and referred by other centers [4,13,19,21,22].

### Post-procedural events

In our series, delayed cerebral ischemia (DCI) occurred in 166 patients (36.3%) and was the most frequent complication. Our results are thus in accordance with the literature.

The incidence of acute re-rupture after coiling embolization of ruptured saccular intracranial aneurysms is between 1.0% to 3.6% [21,25]. Dissecting aneurysms have different etiological and anatomical characteristics. The recurrence of SAH is not uncommon with a rate of 40% specifically for patient treated conservatively [16,21,25]. In the present series, two patients suffered from an early rebleeding. One was a saccular intracranial aneurysm with an acute re-rupture probably due to an incomplete occlusion during the first EVT. The second is a dissection treated by stenting. Our results compare favorably with the literature (0.4%).

### Clinical outcomes at discharge

The ISAT study showed 74.6% of modified Rankin Scales (mRS) between 0 – 2 and 25.4% of mRS between 3 – 6 which are like our results even if we have more patients without any symptom (42%) and more fatalities (17.8%) compared to ISAT (20% and 7.5% respectively) [7,8].

As illustrated in ***Figure 6***, a significant proportion of patients at discharge are in a worse clinical condition than immediately after EVT. Post-procedural events like DCI, intracranial hypertension or epileptic seizure may explain this worsening.

### Limitations

Our monocentric retrospective study has several limitations despite the fact that our database was prospectively maintained. Some data could have been collected to provide interesting information such as the aneurysm neck size, patient risk factors, the severity of the bleeding on CT scan, the detailed presentation of SAH. On the other hand, mid- and long-term results were not evaluated in the present study. Aneurysm recanalization and late rebleeding are significant issues and could be part of a complementary study to evaluate long-term results of EVT of ruptured IA [5,12,17,25]. Finally, data concerning patients treated by surgical clipping were not evaluated.

## Conclusion

This study shows that EVT is safe and effective for patients with ruptured intracranial aneurysms, especially when high practitioner experience and high-volume centers are available. However, even if SAH management has improved over the years, associated complications still lead to significant neurological impairment in some patients. Further research on these topics is mandatory to improve the clinical course of these patients.

## Appendix

**Table:**

EVT FAILURES				

PATIENT GENDER/AGE	WFNS BEFORE EVT	EVD	ANEURYSM CHARACTERISTICS	REASON OF THE EVT FAILURE

**F/36**	2	No	PICA, small	Unreachable

**M/57**	2	Yes	ACom, large	Risk of vascular occlusion

**M/78**	4	Yes	ACom, small	Carotid stenosis

**F/56**	1	Yes	ACom, small	Coil instability

**F/54**	2	Yes	PICA, small	Risk of vascular occlusion

**F/43**	5	Yes	ACom, small	Risk of vascular occlusion

**F/84**	1	Yes	PCom, large	Coil instability

**F/74**	2	Yes	ACom, small	Carotid stenosis

**F/50**	1	Yes	ACom, large	Coil instability

**M/56**	1	Yes	MCA, small	Coil instability

**M/53**	1	No	PCom, small	Too small aneurysm size
